# Understanding the explanatory model of the patient on their medically unexplained symptoms and its implication on treatment development research: a Sri Lanka Study

**DOI:** 10.1186/1471-244X-8-54

**Published:** 2008-07-08

**Authors:** Athula Sumathipala, Sisira Siribaddana, Suwin Hewege, Kethaki Sumathipala, Martin Prince, Anthony Mann

**Affiliations:** 1 Department of Health Services and Population Research, Institute of Psychiatry, Kings College, De Crespigny Park, London SE5 8AF, UK; 2 Institutes of Research and Development, 762/4B Pannipitiya Rd, Battaramulla 10120, Sri Lanka; 3 Centre for Biomedicine and Society, Kings College, Strand, London WC2R 2LS, UK

## Abstract

**Background:**

Patients with medically unexplained symptoms (MUS) are often distressed, disabled and dissatisfied with the care they receive. Illness beliefs held by patients have a major influence on the decision to consult, persistence of symptoms and the degree of disability. Illness perception models consist of frameworks to organise information from multiple sources into distinct but interrelated dimensions: identity (the illness label), cause, consequences, emotional representations perceived control and timeline.

Our aim was to elicit the illness perceptions of patients with MUS in Sri Lankan primary care to modify and improve a CBT intervention.

**Method:**

An intervention study was conducted in a hospital primary care clinic in Colombo, Sri Lanka using CBT for MUS. As a part of the baseline assessment, qualitative data was collected using; the Short Explanatory Model Interview (SEMI), from 68 patients (16–65 years) with MUS. We categorised the qualitative data in to key components of the illness perception model, to refine CBT intervention for a subsequent larger trial study.

**Results:**

The cohort was chronically ill and 87% of the patients were ill for more than six months (range six months to 20 years) with 5 or more symptoms and 6 or more visits over preceding six months. A majority were unable to offer an explanation on identity (59%) or the cause (56%), but in the consequence domain 95% expressed significant illness worries; 37% believed their symptoms indicated moderately serious illness and 58% very serious illness. Reflecting emotional representation, 33% reported fear of death, 20% fear of paralysis, 13% fear of developing cancer and the rest unspecified incurable illness. Consequence and emotional domains were significant determinants of distress and consultations. Their repeated visits were to seek help to alleviate symptoms. Only a minority expected investigations (8.8 %) or diagnosis (8.8%). However, the doctors who had previously treated them allegedly concentrated more on identity than cause. The above information was used to develop simple techniques incorporating analogies to alter their perceptions

**Conclusion:**

The illness perception model is useful in understanding the continued distress of patients with persistent symptoms without an underlying organic cause. Hence it can make a significant contribution when developing and evaluating culturally sensitive patient friendly interventions.

## Background

Worldwide, patients presenting with medically unexplained symptoms (MUS) are common and their health consequences are not a peculiarity to one culture [1-4]. These patients place a heavy burden on the health care delivery system in terms of disproportionate consumption of health resources [5,6], through repeated consultations from specialist and alternative carers [7-9]. Symptomatic investigations and treatments are largely ineffective and iatrogenic [10,11].

Categorising patients with MUS into existing psychiatric diagnoses is unsatisfactory [12]. Only half of the patients with MUS meet the criteria for mood and anxiety disorders [13,14]. Many have neither physical nor mental illness and may simply be distressed [15-17]. This leaves a substantial proportion of patients who do not fit into conventional diagnostic categories and lack a clear indication for treatment although they have significant distress [18-20].

Thus, the traditional psychiatric diagnosis and its underlying epistemology, the biomedical model, hinder both the understanding of this complex problem and its management [21]. Also, the conceptual borders between somatic presentations and mental disorders are ill defined and inconsistent [20]. Looking for an alternative model to understand these patients will prove helpful in managing their distress and disability due to continued somatic symptoms. Common Sense Model (CSM) is one such alternative [22].

Illness beliefs held by patients influence their decision to initiate a consultation as well as the persistence of symptoms and the degree of disability [23-25]. Illness perception models consist of theoretical frameworks to organise information on patients beliefs and expectations into distinct but interrelated dimensions: identity (the illness label), cause, consequences, emotional representation, timeline and perceived control [14,26-28]. These are strong predictors of health outcomes [29].

Even though varying names such as illness perception model, self-regularity model, parallel process model, explanatory model are used, all are derived from the Common Sense Model (CSM) [26]. It describes how an individual constructs an internal representation of what is happening when they experience physical or psychological symptoms [26].

We collected qualitative data for assessment of patients with MUS in a pilot randomised controlled trial (RCT) that tested the effectiveness of CBT [9]. After the pilot RCT [9], we analysed this qualitative data to refine the CBT intervention for the second larger RCT [30]. In this paper, we present how this qualitative data was categorised into dimensions in CSM for further development and modification of the CBT intervention. This step was important as conceptual clarity was needed regarding (a) what the treatment is designed to accomplish and (b) the mechanisms through which a treatment might achieve the required changes [18].

## Methods

### Setting and patient recruitment

The study was conducted in a general out patients' clinic providing primary care, at Sri Jayewardenepura General Hospital in Colombo, Sri Lanka, where patients initiated their own visits, without appointments. This clinic served as a primary care facility. The primary care doctor identified patients between 16–65 years with repeated consultations for MUS and referred them to the research psychiatrist (AS). Detailed description of the study is presented elsewhere [9].

MUS was defined as; 'incompatibility of the clinical presentation with a known physical illness and/or absence of relevant positive physical signs and/or laboratory investigations not supporting a diagnosis of a physical illness' [8,9].

### Study sample

A sample of 68 patients with five or more MUS of any duration was recruited (34 each into two arms) after obtaining written informed consent. Exclusion criteria were dementia, alcohol dependence, psychosis, active suicidal ideation and those currently on psychiatric treatment.

The data presented here was collected at baseline as the assessment part of a RCT providing CBT for patients with MUS in Sri Lanka [9]. Ethical clearance was obtained from the Institute of Psychiatry, UK and approval from the Board of Management of the Sri Jayewardenepura Hospital.

### Instruments

There were two main approaches which were considered, use of closed questionnaires or open-ended interviews to gather information on illness perceptions [31]. Illness perception questionnaire's (IPQ-R) which fell into the first category were not appropriate as it included a fixed rage of responses from which patients had to identify one close to his perception [25,31]. Patients from a different culture may have difficulty in selecting a choice even if similar, because different meanings may be conveyed after translation of the instrument.

Open-ended interviews can be used with much more flexibility, to gather health belief data [32,33]. Bhui & Bhugra [31] compared the Short Explanatory Model Interview (SEMI) and the Explanatory Model Interview Catalogue (EMIC) [32,33] and discussed the advantages of the SEMI. As the name implies it is short and simple and the patient can provide short answers that are easily coded [31].

SEMI elicits assumptions, beliefs and thoughts about their symptoms and its causes, fears about their future, reduction in usual function, increase in dysfunctional behaviour, the expectations of the patients and their satisfaction with care. It is written in simple language. [see additional file 1]

Each section of the interview is designed to stand-alone which allows flexibility in the order of questioning and the interviewer can omit parts of the SEMI according to the study objectives.

### Translation and adaptation of SEMI

AS, was trained to use this interview in English. SEMI and the coding manual were translated to Sinhalese and back translated by two bi-lingual psychiatrists and SH independently. Translated scripts were compared and discussed to find the best conceptual equivalence in Sinhala. Back translated scripts were compared with the original version and consensus was reached on a satisfactory linguistic equivalence.

Sinhalese SEMI was piloted among 10 patients who were not participants in this study. Their responses were coded using the original manual to modify and accommodate the responses generated.

### Data collection

AS interviewed all 68 in the study using the SEMI. The interviews were not audio recorded as it was felt it would be culturally too sensitive and could adversely affect the study participation. Therefore interviews were noted verbatim and another psychiatrist independently co-rated 13 randomly selected patients for reliability and comparability.

SEMI was carried out as the assessment part of the intervention. Therefore the information elicited through the interview was noted then and there to be used in the intervention, rather than transcribing it later. The reason being for most patients the first session was conducted on the same day after assessment. However, because the questions were short and structured, and the answers short it was possible to accurately record verbatim without limiting the pace of the interview. For each question some patients provided single worded replies while some others elaborated their answers. The answers to each question was recorded, after which the next question was posed. Each interview took 15 minutes on average.

AS and SH coded the SEMI scripts independently, using the coding manual after each interview. [see additional file 2] Subsequently the coding by AS and SH carried out on the individual responses documented in the scripts were compared for agreement. Disagreements were discussed and re-coded. This was repeated for all 68 patients.

All data un-coded (symptoms) and coded (perceived causes – internal, external etc) were entered in to a SPSS spread sheet.

### Data analysis

#### Justification of using a combined qualitative and quantitative approach

Research on doctor-patient communication is best done incorporating qualitative and quantitative methods [34].

#### Analysis

Data entered in to SPSS files were analysed using descriptive statistics. Some of the qualitative data was categorised and analysed by hand. No other specific qualitative data analysis software was used. This was a semi structured interview with detailed coding manuals. Due to the specific nature of the interview and coding, we did not have to carry out any additional thematic analyses either.

## Results

### Cohort characteristics

The study group consisted of 48 females and 20 males. Of the females, 12 were unmarried, 28 married, six widowed and two separated. Eight males were married and the rest single. The mean age of the males was 32.4 (SD 10.8), the females was 40.0 (SD 13.7) and the total group was 38.4 years (SD13.4). The cohort was chronically ill, only 12 reported having symptoms for less than six months. Others had symptoms from 6 months to 20 years. Nature and frequency of the presenting symptoms are reported in table 1.

**Table 1:** Nature and frequency of the presenting complaints

**Presenting complaint**	**Number of patients (%)**
Low backache	37(54%)
Chest pain (including back of the chest)	27(40%)
Pain in the limbs	26(38%)
Abdominal pain (including lower abdominal pain)	15 (22%)
Headache	23(34%)
Pain in the joints	21(31%)
Numbness in various body parts	20(29%)
Fatigue	19(28%)
Bloating of the abdomen (puffiness)	14(21%)
Faintish feeling	9(13%)
Loss of appetite	7(10%)
Burning sensation over various body parts	8(12%)
Sleep disturbance	5(7%)
Pain along the spine	3(4%)
Pain in other parts of the body not listed above	26(38%)

### Quantitative and qualitative data arranged under the dimensions in CSM

#### What is it (identity)?

The open-ended question in the SEMI was, 'what do you call these problems (symptoms)? Patients were probed with questions such as 'If you had to give them names what would they be?'

A majority, 46 (59%) participants could not offer any 'diagnosis' or a label for their complaints or symptoms. A physical diagnosis was provided by 14 (21%), and non-specific terms indicating a physical aetiology by 13 (19%). Only one participant gave a psychological diagnosis.

Some common examples were;

'Cancer',

'Excessive 'kapha' (ayurvedic concept referring to phlegm/secretions)',

'I can't identify anything specific, it's too difficult to explain, may be a problem with the head',

'Amoebiasis',

'Indigestion',

'Increased blood pressure and haemorrhoids',

'Arthritis',

'Gastric',

#### Why has it happened (cause)?

The open-ended question in the SEMI was 'why do you think these problems started when they did?

The majority, 38 (56%) of participants did not offer a specific cause for their symptoms. 18 (26%) mentioned a cause relating to the internal world, 10 (15%) to the social world and one (3%) to the natural and super-natural world. Details of the 'cause/s' for the symptoms given by the participants according to the SEMI coding (internal, social, natural and super-natural worlds) are provided in the table 2.

**Table 2:** Explanations given by the patients for symptoms categorised according to the SEMI coding manual.

**Perceived cause**	**Frequency**	**Percentage**
**Internal world (originating in the body or mind)**	**18**	**26.4**
**Mechanical**-damage/blockage/abnormal function	6	8.8
Examples; *'Happened after the child birth; caesarean section',' Because of an injury to my leg 5 years back', 'Gastritis',' Illness of nerves', 'A fish bone or a metal pin stuck in the throat', 'I have swallowed a metal pin in 1950','a poison has got into my body'*,		
**Psychological**/Emotional-worry/fear/upset/sad/lazy	5	7.4
Examples;*'Because I breast fed my child while another child was watching', 'Brother got paralysed at the age of 37 years and I think about it'*		
**Substance abuse**-smoke/alcohol/	1	1.5
Examples; *'Because of chewing betel', 'Did not eat my meals on time and neglected myself'*,		
**Fatigue**- run down/weak spot/non specific stress	2	2.9
**Ageing**	2	2.9
**Imbalance**-diet/vitamin/lack of blood/hot cold	2	2.9
Examples;*'Because of the Vatha' (ayurvedic concept referring to wind or gas) 'Due to too much heat in the body'*,		

**Natural world**	**1**	**1.5**
**Weathe**r-rain/damp/sun/cold	1	1.5

**Social world**	**10**	**14.8**
**Wrong action by health professionals**	3	4.4
**Delay in seeking help**	1	1.5
**Discrimination**	1	1.5
**Money**	1	1.5
Examples;*'Because I was not successful in life'*		
**Work related**	4	5.9
Examples;*' Because I was in the catering field I developed anorexia for food'*,		

**Supernatural world**	**1**	**1.5**
**Supernatural or Magic by a human agency**	1	1.5
Examples;*'An evil spell cast by somebody else,' 'Due to de-merits of an earlier birth'*		

**Any other – **(vague answers that did not fit into any of above)	**13**	**19.1**
**Unable to say**	**25**	**36.8**
*'No idea', 'Don't know'*		

**Total**	**68**	**100.0**

#### What effects will it have(consequences)?

The open-ended question in the SEMI was, 'how serious are your problems?'

95% had clear opinion on the seriousness; 37% believed their symptoms indicate moderately serious illness and 58% very serious illness.

When they were requested to elaborate, some responses given were;

'I will develop chest pain and tumours',

'I may get bed-ridden'

'I may need major surgery'

'I will die and if so what will happen to the children'?

'Will be grave, I am scared because the severity of symptoms may increase',

'I won't be able to conceive a child',

'It may be a cancer, because doctors are saying nothing is wrong and the symptoms are not responding to treatment. My mother also died of cancer',

'It looks like an incurable illness, I might remain ill everyday in the future',

'It is the beginning of a serious illness'

'Will I suffer hemiplegia/stroke because my mother also died of a stroke and I may not be able to walk?'

'I fear a fainting attack while crossing the road'

'Start of a serious illness, with time this illness will become severe'

'I will be always a sick person',

'Won't live much longer'

#### Emotional representation

The open-ended question used was 'what do you fear most about these problems' (symptoms)?

Reflecting emotional representation, 66 (97%) expressed having some form of a fear, only two denied any concern. 33% reported fear of death, 20% fear of paralysis, 13% fear of developing cancer and the rest unspecified incurable illness.

#### How long will the symptoms last, will it recur (timeline)?

SEMI did not contain any specific question on this dimension. Nevertheless as reported in previous paragraph, majority had fear of potential serious complications in the future, which implies that they have perceived their symptoms as chronic or recurring.

#### What can I do to make it go away; cure or control?

The questions that covered this dimension were, 'what you can do about it, who you saw about it, and what did your doctor do'. Most, had consulted allopathic doctors 63 (92%), but in addition 33 (48%) sought help from alternative practitioners. They had had turned to family; 60 (88%), friends; 35 (52%) and clergy; 3 (4.4 %) for advice. A majority, 34 (50%) specifically wanted the doctors to make them better, 9 (13%) wanted advice and explanation, 8 (12%) medication, 6 (9%) a diagnosis while another 6 (9%) requested further investigations. However, none had requested a referral to a specialist. Thirty-two participants reported at least one hospital admission over the preceding six months before the assessment. The mean number of visits to the health care providers was 7 (SD 5.2, range 0–30). General practitioners were visited by 63 (92%), general physicians by 39 (57%) and 33 (48%) participants went to alternative practitioners. Various other categories of specialists were visited by between 3–10 participants on their own initiative. Only two patients had visited psychiatrists.

Examples of recorded responses given to the question as to what should they do about their symptoms were;

'I should take bed rest',

'I should rest',

'Sleep',

'I should find out the illness I am having',

'It should be investigated',

'Should take medications',

'Should take treatment from doctors',

'Should apply medicinal balm'

### Patient's perceptions and interpretation on the encounter with the doctors

The questions were; What do/did you hope to gain from seeing doctors? What did you expect/want the doctors to do? What did the doctors tell you and what did the doctors do about your problems?

A majority, 34 (50%) specifically wanted the doctors to make them better but did not clarify how, 9 (13%) wanted advice and explanation, 8 (12%) medication, 6 (9%) a diagnosis while another 6 (9%) expected further investigations. However, none had expected a referral to a specialist.

Forty participants (59%) reported that their doctors mentioned a non-specific illness indicating underlying organic aetiology. Nineteen participants (28%) said they were given a specific physical diagnosis and 24 (35.2%) had been told that there was no illness. Doctors did not give any explanation for 7 (10%), 11 (16%) were advised to ignore their illness, another 9 (13%) participants were told 'not to be frightened'. Four (6%) were told that 'every thing was in their mind' implying a psychological aetiology. Overall fifty-seven (83%) had many different explanations given.

Examples included;

'No illness, no abnormality found in the reports',

'Not to draw water from the well',

'Not to be afraid',

'Gastric illness has gone to your head',

'You are too obese',

'This is an illness in the mind and to get rid of it we will do some tests',

'Tell the gods/spirits',

'It is a psyche illness, you are thinking too much'.

### An example of a narrative account of a patient to clarify the model

The following is a narrative account of an explanatory model interview with a patient, which will be informative for individual patient management. Similar summaries were provided on each patient for use in CBT sessions by the primary care doctors in the second RCT.

"The patient presented with abdominal pain, headache, chest pain, backache, pain along right upper limb and numbness of fingers of 5 years duration. She was unable to give an exact name (a label) and possible cause of the illness, but believed that working too much may be a reason. She also believed that her husband was responsible for her illness, as he never helped in housework (cause; social world). She perceived her illness to be very serious and suspected it might be a cancer (consequence and also an indirect indication of label). She had been to eight different doctors of different specialities. They had conducted ECG, s X-rays of chest and spine, blood tests, urine tests and many other tests she was unable to describe. All of these had been normal. Most doctors told her that there was 'nothing wrong'. However, she was unhappy (emotional reaction) as the symptoms persisted and worried (emotional reaction) she may never be cured (time line – chronic). As a result of these symptoms, she was unable to do any housework and had given up her permanent job as a cashier (disability)."

## Discussion

Qualitative data; information elicited by the SEMI categorised into "illness representation model" revealed that a majority were unable to offer an explanation on the identity (59%) or the cause (56%), but in the consequence domain 95% had significant illness worries and in the emotional representation domain, 97% had a significant fear of; death, paralysis, cancer or unspecified incurable illness. Consequences and emotional domains were significant determinants of distress and consultations. A majority offered neither a Western biomedical label or a cause, nor any locally influential cultural label or attribution [35]. It is unlikely that the inability to offer a label or a cause can be explained based on illiteracy, as Sri Lanka has a literacy rate over 90% [36]. The repeated visits by the patients were to seek help in alleviating continued symptoms and most wanted the doctors to 'make them better', suggesting external locus of control. Only minority expected investigations (8.8 %) or diagnosis (8.8%). Contrary to patients' expectations, self-reports indicated that, the doctors appeared to have concentrated more on identity and cause. Similarly it has been shown that even in the west, GP's often misperceive what patients seek, and their treatment decisions are more related to their perception of patients' wishes than to patients' actual wishes [37-40]. It appears that the patients' expectations in our study too, were not met by their carers. This could be the possible reason for repeated visits to many different categories of care.

The somatic interventions used by doctors has been widely attributed to patients belief that symptoms are caused by physical disease [41,42]. However in our study majority did not offer an explicit physical cause or a diagnosis as the label. But, the responses given to possible consequence, such as 'it may be a cancer' and also the responses to fear domain (that it may be cancer or they may become paralysed) possibly indicates a perceived physical basis. Therefore it has to be noted that even though the dimensions are distinct they are interrelated and should be taken in context.

Even though a direct comparison cannot be made, it is useful to discuss what was reported on primary care patients presenting with physical symptoms in the USA [43]. In their study, 81% expected a diagnosis (an explanation for the symptom), 63% prognosis, 54% investigations, 66% medication, and 45% referral to a specialist. Primary care presentation by patients were prompted by concern about the cause and prognosis and less so by the severity of the symptoms.

To our knowledge there are no studies carried out in the UK using SEMI on patients exclusively with medically unexplained symptoms. However, the original validation study among patients with common mental disorders, presenting complaints was mostly non-specific physical symptoms [57]. Even though none of the patients in our study volunteered psychological symptoms in contrast 13.9% of Indo-Asians, 6.7% of white British, 8.9% of Afro-Caribbean in the above [57]. Although most patients (58.8%) in our study were unable to give a specific biomedical diagnostic label for their complaints it was only 30.6% among Indo Asians, 19.7% Afro-Caribbeans in UK, 13.3% White British [57] Significantly, relation to the super-natural world is a particularly interesting one from a cross-cultural point of view as 11.2% Asians in London believed in spells and black magic/obai.

There is limited work reported from the developing world on the illness perception of patients with MUS [44,45]. A South Indian study using SEMI for MUS, contrary to our findings, reported that majority of patients expressed strong beliefs about physical nature of their complaints and 42% believed black magic as a cause [46]. However, as in our study the majority (98%) believed symptoms were serious and feared disability or death.

### Implications of the findings on refining CBT intervention

Knowledge of the likely factors leading to the clinical problem or dysfunction, the processes involved, and how these processes emerge or operate, can contribute to the treatment development research [18]. Illness perception models of majority of Sri Lankan patients with MUS were overwhelmingly dominated by two explicit factors; the consequence dimension of the cognitive representation and the fear dimension of the emotional representation. It is known that patients with MUS have more negative illness perceptions, and the emotional representation dimension is highly correlated with the consequences dimension [29]. As our narrative highlighted, the patient assumed that her symptoms are indicative of cancer and was worried that she would never be cured. Similarly, in cognitions she made up an assumption that she may have 'possibly developed cancer due to over work'. Hence, during 'cognitive restructuring' these thinking errors were directed at de-linking 'over work' and 'cancer'.

Similarly simple strategies through development of analogies were undertaken to explain why their MUS did not represent a serious life threatening illness, rather than explaining why further investigations are not indicated to find a cause, as the majority did not demanded any (47). The model on individual patients helped the therapist to explore individual variations in different domains within the framework and carryout individual modifications to the therapy based on the specific component unique to them.

Use of SEMI information provided an opportunity for the therapist to understand what the patients expected and didn't, and therefore was helpful in avoiding unnecessary debates with the patient as to whether these symptoms were physical or psychological [48-50]. This was important, as most of the doctors who previously treated these patients had fallen in to that trap: some saying that it was, and others saying that there was no physical illness. Thus, the therapists were able to concentrate on most suitable strategies to provide appropriate cognitive challenge, which would have been different for patients who demand more investigations and diagnosis.

Because the symptoms are unexplained, cognitions can be puzzling and distressing. Therefore providing simple reassurance [30] and 'normalisation statements with explanation on negative test results or on patient's concerns acknowledging the suffering, and providing tangible mechanisms to explain symptoms' were what was needed [51,52]. This information was also useful in developing simple techniques incorporating analogies to deal with their perceptions [47,53]

Cultural differences affect illness behaviour, help seeking, expectations of the patient and perceived quality of care. Therefore, understanding the importance of these elements is paramount in the design of culturally appropriate psychotherapies [54]. Understanding these illness representations will enable a clinician to appreciate suffers response to illness, develop an empathic relationship and communicate his own interpretation and recommendations for treatment more effectively [31]. Gathering such qualitative information is a pre-requisite of developing psychological interventions [55,56].

### Implications for clinicians

Even during a routine clinical practice the doctor could elicit patients' views and interpretation of their symptoms by asking what the patient's thought about their symptoms, the potential consequences, resulting fears and what they expect from the clinician.

Discussing patient's views will enable a clinician to appreciate the patient's response to illness,, to develop an empathic relationship and to communicate his explanation and recommendations for treatment more effectively. By recognising areas where the patient's and the health care providers understanding of the problem are different, the clinician can address these differences. This may result in negotiating a shared model but if the differences are irreconcilable, the clinician can acknowledge them and work with the patient in a manner so as to avoid conflicts and thus maximise the chances of compliance to the clinician's treatment. Empirical evidence suggests that patients are most satisfied where their therapists share their model of understanding distress and treatment [57].

## Strengths and limitations

This study has looked into; the illness perceptions of the patient, which is important but inadequately explored in patients with MUS, particularly in the developing world. However, as we have not used a randomly selected control group of patients in the same out patients' clinic, it is not possible to conclude whether these findings are specific to patients with MUS or is a reflection of Sri Lankan patients in general. As the interviews were not recorded or transcribed there was a potential risk of losing information when verbatim responses are recorded manually [31]. However, the research team decided against audio recording, as it was perceived to be too culturally insensitive. As responses generated by SEMI questions were short, observation and co-rating of a random sample of interviews by another investigator this limitation may have compensated to a large extent.

Relying on patient's information and interpretation to deduce what the doctors believed or advised may have introduced a recall bias. However, these facts reported by the patients reflect what they perceived from the encounters with the doctors.

It could be argued that a further limitation is the nature of the interviewer; in this case a psychiatrist, patients might express themselves differently depending on what they know about the interviewer and the environment in which the study is conducted. Although the interviewer was a psychiatrist (AS) he was also a qualified primary care physician who was previously in charge of the primary care setting where the study was conducted. Due to this reason and the fact that the study was conducted in this primary care out patients' facility, it is unlikely that during the initial assessment this factor significantly affected the study. However after the CBT sessions and latter part of the study, some became aware that the AS was a psychiatrist and we guess some of the drop out during the follow up was likely be due to this factor. Even with some of these limitation, the finding will have implications for clinical practice, as a study of this nature has not been conducted in such a setting previously.

## Conclusion

Understanding of cognitions and its complex interactions with patients' experiences and symptoms by using qualitative data is important when developing and evaluating cognitive behavioural interventions that are culturally sensitive and patient friendly [58,55]. The illness perception model is useful in eliciting such information that contribute to the continued distress of patients with persistent symptoms without an underlying organic cause and in their management.

## Competing interests

The authors declare that they have no competing interests.

## Authors' contributions

AS proposed the initial conceptual framework for the research and was responsible for overall conduct of the project. AS, SS, SH, MP and AM were involved in the protocol design, analysis and or interpretation of data, writing and editing the manuscript. AS and SH contributed to the data collection. AS, SS, SH, KS, contributed to the analysis (and re analyses) of data. AS prepared the first draft. SS, SH, KS, MP, AM contributed to the subsequent editing and agreed on the final version. AS, SS and KS were involved in the revisions made, based on the reviews provided by the journals reviewers.

## Pre-publication history

The pre-publication history for this paper can be accessed here:


